# Evaluating comorbidity scoring systems for flumatinib therapy in chronic myeloid leukemia: a machine learning and SHAP-based predictive analysis

**DOI:** 10.3389/fmed.2026.1849735

**Published:** 2026-05-28

**Authors:** Yuanlan Yang, Yujun Li, Jishi Wang

**Affiliations:** 1 Guizhou Medical University, Guiyang, China; 2 Department of Hematology, People's Hospital of Qianxinan Buyi and Miao Autonomous Prefecture, Xingyi, Guizhou, China

**Keywords:** chronic myeloid leukemia, comorbidity, flumatinib, machine learning, precision medicine

## Abstract

**Background:**

Comorbidities increasingly complicate targeted therapy for chronic-phase chronic myeloid leukemia (CP-CML). This study evaluated three comorbidity scoring systems (CCI, ACE-27, and CIRS-G) to predict molecular responses in flumatinib-treated CP-CML patients.

**Methods:**

Retrospective data from 559 patients (2018–2024) were analyzed. Machine learning algorithms, including XGBoost, were trained to predict 12-month major molecular response (MMR). SHapley Additive exPlanations (SHAP) analysis was employed to interpret non-linear associations and feature interactions.

**Results:**

The XGBoost model demonstrated the highest predictive performance (AUC = 0.852). Integrating CIRS-G into the baseline clinical model provided the most substantial incremental value (ΔAUC = 0.078, *p* = 0.006), outperforming CCI and ACE-27. SHAP analysis revealed a non-linear threshold effect, suggesting that a CIRS-G score ≥ 8 may severely compromise therapeutic efficacy. Furthermore, interaction plots indicated an exploratory model-based association where advanced age and severe comorbidity clustered with lower predicted probabilities, while reduced dose interventions coincided with altered model predictions in this specific subpopulation.

**Conclusion:**

CIRS-G showed the strongest incremental predictive value among the evaluated comorbidity scores in this single-center cohort. Machine learning and SHAP analysis provide exploratory insights for individualized risk stratification, though the reported predictive performance may overestimate prospective validity due to temporal variations and retrospective design.

## 1 Introduction

The introduction of tyrosine kinase inhibitors (TKIs) has fundamentally transformed the management of chronic-phase chronic myeloid leukemia (CP-CML), shifting the disease trajectory from a rapidly fatal condition to a manageable chronic illness (1, 2). Flumatinib is a Chinese-developed second-generation TKI. Flumatinib has demonstrated high efficacy and a favorable safety profile in newly diagnosed CP-CML patients, leading to deep molecular responses and durable disease control (3, 4). Overall survival for CP-CML patients is now in the range of the normal population. This has now shifted the disease demographic to older patients (5). A major clinical concern now is age-related comorbidities. Treating patients with multiple chronic diseases can have a negative impact on the therapeutic outcome due to the alterations in drug pharmacokinetics as well as the increase in adverse drug reactions and the reduction in compliance to prescribed therapies due to less desired outcomes (6–8). All of these factors can influence the overall efficacy of the treatment.

When assessing the impact of concurrent diseases among oncology patients, comorbidity scoring systems are a well-established concept (9, 10). Among these, the most common is the Charlson Comorbidity Index (CCI), which is primarily designed to estimate long-term mortality risk for specific diagnoses (11, 12). In the context of solid tumors, the Adult Comorbidity Evaluation-27 (ACE-27) is often used for a more specific breakdown of the severity of the comorbidities (13). On the other hand, the Cumulative Illness Rating Scale for Geriatrics (CIRS-G) is a better fit for older patients as it focuses on a more integrated physiological approach to different systems (14). However, which of these scales is the most useful in predicting molecular responses in CP-CML patients on TKI treatment, is still unanswered (15). This is also the case in using older statistical techniques that are based on the assumption of linear relationships among different clinical factors and treatment outcomes (16). In particular, this creates an over-simplification of the complex and varied relationships that exist among age, comorbidity burden and drug dosing (17).

Machine learning models utilize advanced analytical techniques that identify non-linear relationships and interactions within the clinical data that can capture very high dimensions (18). Recently, advancements in interpretable artificial intelligence, such as SHAP, have provided these models with additional transparency, allowing researchers to convert complex predictive models into useful clinical data summaries (19, 20). The SHAP analysis explains the clinical data summaries by assessing the marginal contribution of each of the features, and in doing so, the analysis uncovers particular clinically relevant thresholds and synergistic relationships that have predictive value concerning the clinical prognosis of the patients (21).

With this in mind, the main purpose of this study was to analyze and assess the predictive value of the comorbidity indices CCI, ACE-27, and CIRS-G regarding the molecular responses of patients with CP-CML who received flumatinib after 6 and 12 months of therapy. The aim of this research was to integrate comorbidity indices into machine learning models to capture the non-linear effect of comorbidity as an obstacle to the success of a therapeutic intervention, and to provide clinical evidence for a personalized approach to dose adjustment within the framework of SHAP for global and local interpretability.

## 2 Materials and methods

### 2.1 Study design and ethical approval

This retrospective cohort study was conducted at the People’s Hospital of Qianxinan Buyi and Miao Autonomous Prefecture, evaluating clinical data collected between January 2018 and January 2024. The study protocol adhered strictly to the ethical principles outlined in the Declaration of Helsinki. Ethical approval was granted by the Institutional Review Board of the People’s Hospital of Qianxinan Buyi and Miao Autonomous Prefecture (Approval No. 2024-2-S-015). Given the retrospective nature of the study design and the use of anonymized clinical data, the requirement for obtaining written informed consent from the participating patients or their legal guardians was waived by the ethics committee.

### 2.2 Data collection and clinical indicators

Baseline demographic, clinical, and laboratory data were extracted from the electronic medical record system. The comprehensive feature set incorporated into the analysis included demographic variables [age, sex, and body mass index (BMI)], CML-specific prognostic risk stratifications (Sokal, ELTS, and EUTOS scores), and routine laboratory parameters prior to treatment [white blood cell count (WBC), hemoglobin, platelet count, basophil percentage, spleen size, and baseline BCR-ABL1 international scale (IS)]. The initial dose of flumatinib (standard 600 mg daily versus reduced dose) was also recorded.

Prior to treatment initiation, the comorbidity burden of each patient was systematically evaluated using three standardized scoring systems: the Charlson Comorbidity Index (CCI), the Adult Comorbidity Evaluation-27 (ACE-27), and the Cumulative Illness Rating Scale for Geriatrics (CIRS-G).

The primary clinical endpoint for the predictive models was defined as the achievement of major molecular response (MMR) at 12 months. Based on the International Scale (IS), molecular responses were strictly defined as MMR (BCR-ABL1 IS ≤0.1%), MR4.0 (BCR-ABL1 IS ≤0.01%), and MR4.5 (BCR-ABL1 IS ≤0.0032%). The allowed clinical assessment window for the designated time points was ±1 month.

### 2.3 Machine learning model development

For developing and validating predictive models, the data set was split using a random approach into a training data set which comprised 70% of the entire data set, and a validation data set, which comprised 30% of the entire data set. In order to combat the class imbalance problem (in the clinical data set) and to avoid the predictive models learning a bias toward the majority class, a class (sample) weighting approach was utilized during the training of the models. For distance-based algorithms, continuous features were normalized using the min-max scaling approach.

The 12-month MMR was predicted with five different machine learning algorithms: Logistic Regression (LR), Support Vector Machine (SVM with a radial basis function kernel), K-Nearest Neighbors (KNN, with k = 25), a Random Forest (RF with 500 decision trees), and eXtreme Gradient Boosting (XGBoost). Variable-level missingness was less than 5% for all collected features. Missing continuous variables were handled using median imputation, while missing categorical variables were handled via mode imputation. For the XGBoost model, rigorous hyperparameter tuning was conducted exclusively within the training set using a grid search strategy combined with 5-fold cross-validation. The independent testing set was strictly locked and accessed only once for the final performance evaluation to prevent data leakage. The hyperparameter search grid included maximum tree depth (3, 4, 5), learning rate (0.01, 0.03, 0.05), subsample ratio (0.7, 0.8, 0.9), and L2 regularization lambda (0.5, 1.0, 2.0). The optimal configuration determined via cross-validation included a maximum tree depth of 3, a learning rate of 0.03, a subsample ratio of 0.7, a column sample by tree ratio of 0.8, a minimum child weight of 2, and a lambda of 1.

### 2.4 Model evaluation and SHAP interpretability analysis

The discriminative ability of the developed models was primarily evaluated on the testing set using the area under the receiver operating characteristic curve (AUC) and precision-recall (PR) curves. DeLong’s test was used to determine whether the differences in the AUC of the baseline clinical model and the models with certain comorbidity scores were statistically significant. For this purpose, the decision curve analysis (DCA) was used to calculate the net clinical benefit at different threshold probabilities.

To analyze the internal decision process of the optimal XGBoost model, the SHAP (Shapley Additive exPlanations) method was used. For global interpretability SHAP values were used, and this includes analysis of feature importance ranking and non-linear relation, while for local interpretability the analysis included individual waterfall trajectories. Continuous variables were summarized as mean ± standard deviation (or median with interquartile range) and the summary was provided based on the distribution of the variables. They were compared using the student’s *t*-test and Mann–Whitney *U*-test, respectively, while categorical variables were summarized using frequency and percentage. For the categorical variables, the comparison was made using the chi-square test, or in some instances, the Fisher’s exact test. A two-sided *p*-value of less than 0.05 was significant. Quantitative calibration was assessed using the Brier score. All statistical analyses and machine learning modeling were executed using R software (version 4.2.2), utilizing packages including xgboost (version 1.7.5.1), SHAPforxgboost (version 0.1.3), pROC (version 1.18.0) for DeLong implementation, and rmda (version 1.6) for DCA plotting.

## 3 Results

### 3.1 Baseline characteristics and therapeutic outcomes stratified by comorbidity severity

A total of 559 patients with chronic-phase chronic myeloid leukemia (CP-CML) receiving initial flumatinib therapy were included in this analysis. The cohort was divided into mild (*n* = 211) and moderate/severe (*n* = 348) comorbidity groups based on the combination of three comorbidity scoring systems (CCI, ACE-27, and CIRS-G). The demographic and clinical characteristics at baseline are shown in Table 1. The \textit{moderate/severe} group was significantly older (56.7 ± 12.9 vs. 43.6 ± 12.2 years, *p* < 0.001) and had a slightly higher median white blood cell count (124.8 vs. 116.3 * 10^9/L, *p* = 0.034) compared to the \textit{mild} group. There was no significant difference in the distribution of patients categorized as high-risk by Sokal, ELTS, and EUTOS scores (all *p* > 0.05); therefore, both groups had a similar baseline disease burden. Concerning the first line treatment, fewer patients in the moderate/severe group received the standard 600 mg dose of flumatinib compared to the mild group (83.0% vs. 93.4%, *p* < 0.001). A detailed cross-tabulation further illustrating the empirical dose assignment patterns stratified by chronological age and CIRS-G severity is provided in Supplementary Table S1.

**Table 1:** Baseline demographic and clinical characteristics of CP-CML patients treated with flumatinib.

Clinical indicators	Total cohort (*n* = 559)	Mild comorbidity (*n* = 211)	Moderate/severe comorbidity (*n* = 348)	*p*-value
Demographics				
Age (years), mean ± SD	51.8 ± 14.1	43.6 ± 12.2	56.7 ± 12.9	<0.001
Sex (male), *n* (%)	301 (53.8)	111 (52.6)	190 (54.6)	0.647
BMI (kg/m^2^), mean ± SD	24.2 ± 3.4	24.1 ± 3.4	24.3 ± 3.4	0.476
CML prognostic scores				
Sokal score (high risk), *n* (%)	123 (22.0)	45 (21.3)	78 (22.4)	0.764
ELTS score (high risk), *n* (%)	101 (18.1)	36 (17.1)	65 (18.7)	0.630
EUTOS score (high risk), *n* (%)	72 (12.9)	30 (14.2)	42 (12.1)	0.462
Laboratory parameters				
WBC ( $\times 10^{9}$ /L), median [IQR]	121.3 [88.0–167.1]	116.3 [84.2–155.8]	124.8 [90.8–170.9]	0.034
Hemoglobin (g/L), mean ± SD	109.0 ± 23.4	108.8 ± 23.5	109.1 ± 23.4	0.886
Platelets ( $\times 10^{9}$ /L), median [IQR]	457.0 [383.0–517.5]	460.0 [391.5–522.5]	450.0 [376.8–513.5]	0.459
Basophils (%), mean ± SD	4.2 ± 2.1	4.3 ± 2.0	4.1 ± 2.1	0.353
Spleen size (cm), mean ± SD	4.6 ± 2.8	4.7 ± 2.8	4.6 ± 2.8	0.819
Baseline *BCR-ABL1* IS (%), mean ± SD	64.7 ± 15.3	65.5 ± 16.5	64.2 ± 14.6	0.338
Comorbidity scoring systems				
CCI score, median [IQR]	2.0 [1.0–3.0]	1.0 [0.0–1.0]	2.0 [2.0–3.0]	<0.001
ACE-27 score ( ≥ 1), *n* (%)	381 (68.2)	86 (40.8)	295 (84.8)	<0.001
CIRS-G total score, median [IQR]	5.0 [3.0–8.0]	3.0 [1.0–4.0]	7.0 [6.0–10.0]	<0.001
Treatment regimen				
Standard dose (600 mg), *n* (%)	486 (86.9)	197 (93.4)	289 (83.0)	<0.001

CP-CML, chronic phase chronic myeloid leukemia; SD, standard deviation; IQR, interquartile range; BMI, body mass index; WBC, white blood cell; IS, international scale; CCI, Charlson Comorbidity Index; ACE-27, Adult Comorbidity Evaluation-27; CIRS-G, Cumulative Illness Rating Scale for Geriatrics.

Continuous variables with normal distribution were compared using Student’s *t*-test and are presented as mean ± SD. Continuous variables with non-normal distribution were compared using the Mann–Whitney *U*-test and are presented as median [IQR]. Categorical variables were compared using the Pearson’s Chi-square test or Fisher’s exact test and are presented as n (%).

**Figure 1:**
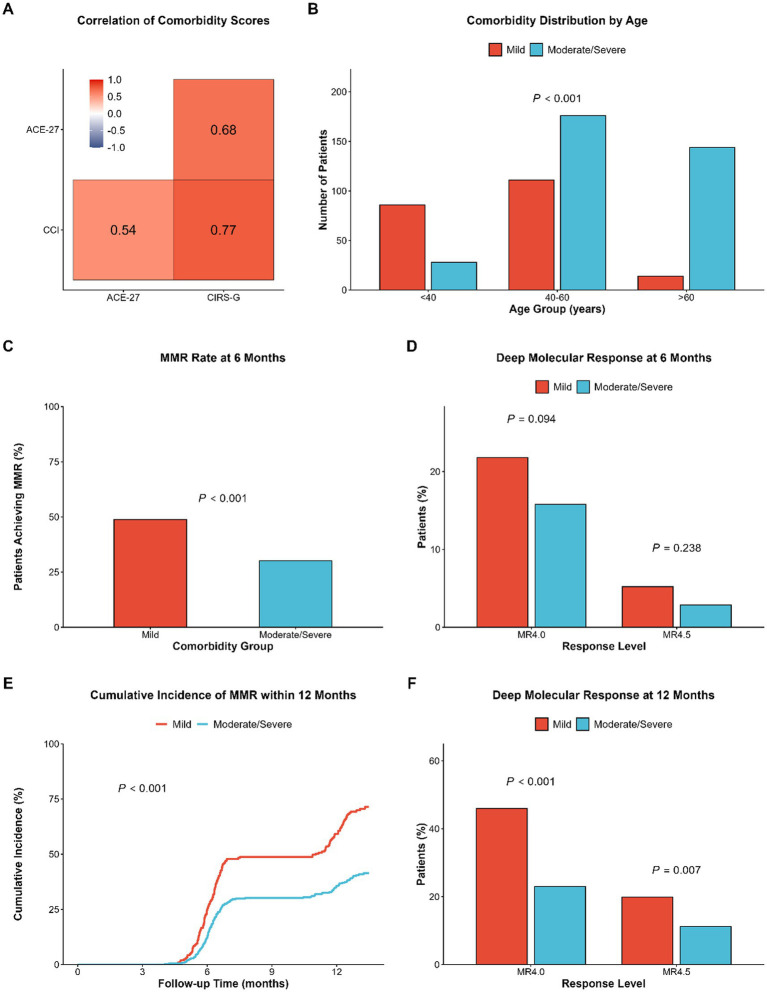
Clinical characteristics and molecular responses stratified by comorbidity severity. **(A)** Heatmap showing the correlation among three comorbidity scoring systems (CCI, ACE-27, and CIRS-G). **(B)** Distribution of comorbidity severity across different age groups. **(C,D)** Proportion of patients achieving major molecular response (MMR) and deep molecular response (MR4.0, MR4.5) at 6 months. **(E)** Cumulative incidence of MMR within 12 months. **(F)** Proportion of patients achieving MR4.0 and MR4.5 at 12 months. Overall, the analysis suggests that moderate-to-severe comorbidities may be associated with lower molecular response rates in CP-CML patients.

The associations among the three comorbidity scoring systems and their influences on clinical outcomes were further analyzed (Figure 1). The CCI, ACE-27, and CIRS-G scores were positively associated with one another (Figure 1A). Demographic data indicated a possible age-related imbalance in comorbidity burden. The count of patients with \textit{moderate/severe} comorbidities was notably high in the cohort aged above 60 years (144 vs. 14, *p* < 0.001; Figure 1B).

The two groups were measured to see what therapeutic outcomes were achieved after 6 months and after 12 months. The results show that there is a statistically significant difference in the 6 month MMR rates for the moderate/severe group and mild group (30.1% vs. 48.8%, *p* < 0.001; Figure 1C). However, the groups did not achieve statistical significance when looking at deep molecular response, MR4.0 and MR4.5, after 6 months (*p* = 0.094 and *p* = 0.238, respectively; Figure 1D). Analysis of the cumulative incidence shows a gap in MMR at 12 months that is statistically significant (*p* < 0.001; Figure 1E), and shows that by 12 months, the moderate/severe group has a statistically significant difference in deep molecular response (MR4.0: 22.9% vs. 45.9%, *p* < 0.001; MR4.5: 11.2% vs. 19.9%, *p* = 0.007; Figure 1F). The results indicate that having moderate to severe comorbidities is a predictor for long-term poor response at the molecular level for CP-CML patients taking flumatinib.

### 3.2 Construction and validation of machine learning models for predicting 12-month molecular response

Building upon the observation that comorbidity burden was associated with therapeutic outcomes, we subsequently incorporated these comorbidity scoring systems into baseline clinical characteristics to construct machine learning models. The aim was to assess their proficiency in forecasting the achievement of major molecular response (MMR) at the 1-year mark. Five representative machine learning techniques were benchmarked and evaluated, namely, XGBoost, Random Forest, Support Vector Machine (SVM), Logistic Regression, and K-Nearest Neighbors (KNN).

**Figure 2:**
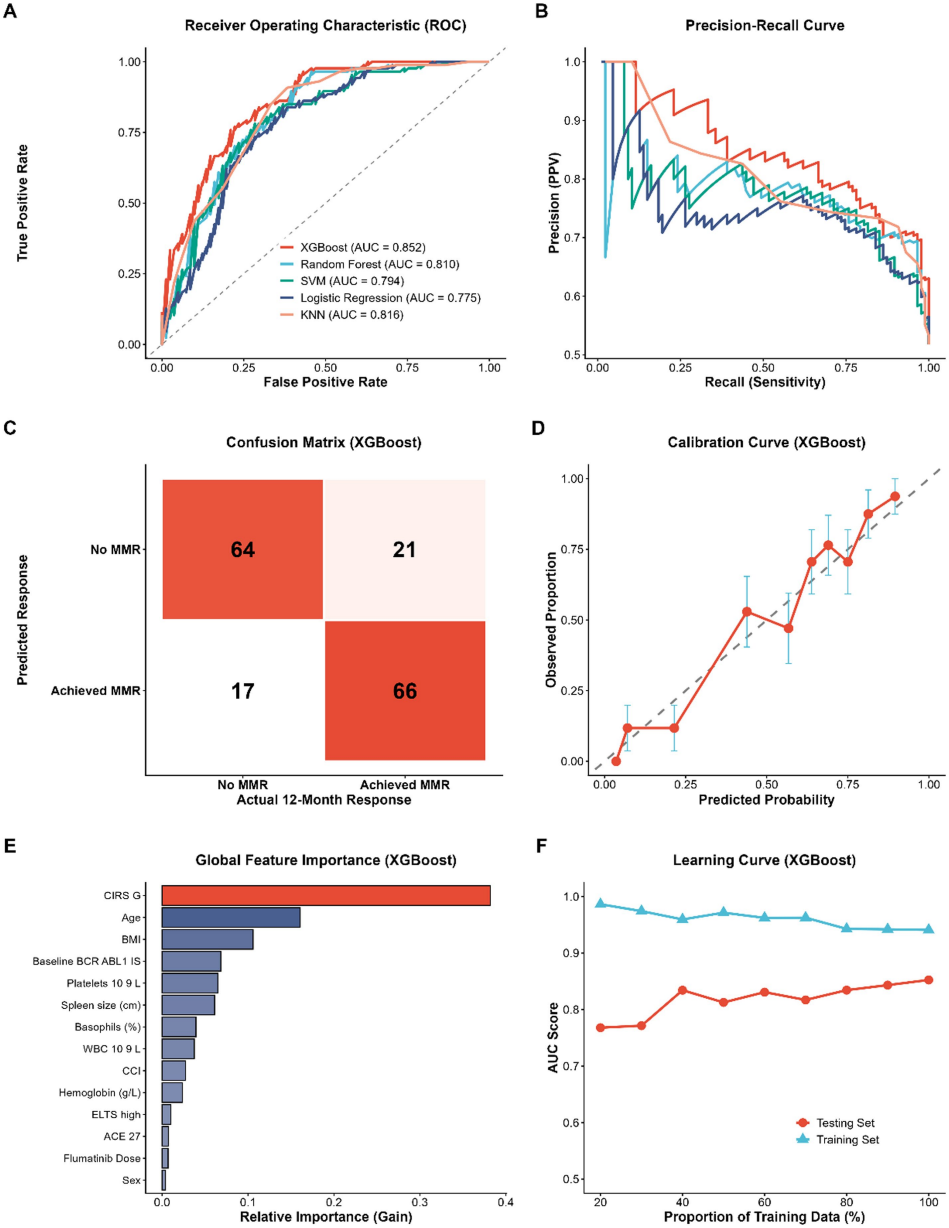
Construction and evaluation of machine learning models for predicting 12-month MMR. **(A,B)** Receiver operating characteristic (ROC) and precision-recall (PR) curves evaluating the discriminative ability of five machine learning algorithms. **(C)** Confusion matrix showing the classification results of the XGBoost model on the testing set. **(D)** Calibration curve indicating the agreement between predicted probabilities and observed frequencies for the XGBoost model. **(E)** Global feature importance ranking based on the XGBoost algorithm (Gain metric). **(F)** Learning curve showing the area under the curve (AUC) across varying proportions of training data. These results indicate that the XGBoost model may provide favorable predictive performance.

For the independent testing set, predictive performance was assessed for each model using Receiver Operating Characteristic (ROC) and Precision-Recall (PR) curves (Figures 2A,B). Findings showed the collective tree-based ensemble algorithms to have good discriminative power. Specifically, the XGBoost model yielded the highest Area Under the Curve (AUC) of 0.852 (95% CI: 0.813–0.891), followed by KNN (AUC = 0.816, 95% CI: 0.771–0.861) and Random Forest (AUC = 0.810, 95% CI: 0.764–0.856), whereas the conventional Logistic Regression model exhibited a relatively lower AUC of 0.775 (95% CI: 0.725–0.825) (Figure 2A). Furthermore, the PR curve showed XGBoost’s performance to be good when considering the satisfactory identification of patients MMR achieved because of a good recall-precision balance (Figure 2B).

For further illustration of the optimal model’s classification power, a data-driven threshold, determined via Youden’s Index strictly on the training set, was applied to yield a confusion matrix for the XGBoost algorithm on the independent testing set (Figure 2C). In the testing cohort of MMR, the model was able to identify 64 cases of non-MMR and 66 cases of MMR, while failing to do so in 21 and 17 cases, respectively, which shows good classification accuracy. Additionally, the calibration curve demonstrated that the predicted probabilities generated by the XGBoost model were closely aligned with the observed clinical frequencies along the diagonal line, which was further supported by a low Brier score of 0.142, suggesting reliable quantitative calibration for potential individualized risk estimation (Figure 2D).

To examine some of the drivers of the XGBoost model, global feature importance was estimated with the Gain metric (Figure 2E). This analysis indicated the feature with the highest contribution (Gain = 0.382) was the CIRS-G score. Other clinical variables that made it to the top were age (Gain = 0.161), BMI (Gain = 0.106), and baseline BCR-ABL1 IS (Gain = 0.068). CIRS-G even outperformed traditional prognostic metrics like the ELTS score and spleen size, implying that the dominant feature of the CIRS-G score may even be of aid when TKI therapy outcomes are predicted. Lastly, the learning curve for the XGBoost model was plotted to evaluate training stability (Figure 2F). When the proportion of the training data was increased, the AUC (Area Under Curve) of the training data slightly decreased and that of the testing data rose to just under 0.85. This trend indicates the model was able to successfully reduce overfitting to a minor degree and showed a good amount of generalization.

### 3.3 Global interpretability and non-linear threshold analysis using SHAP

**Figure 3:**
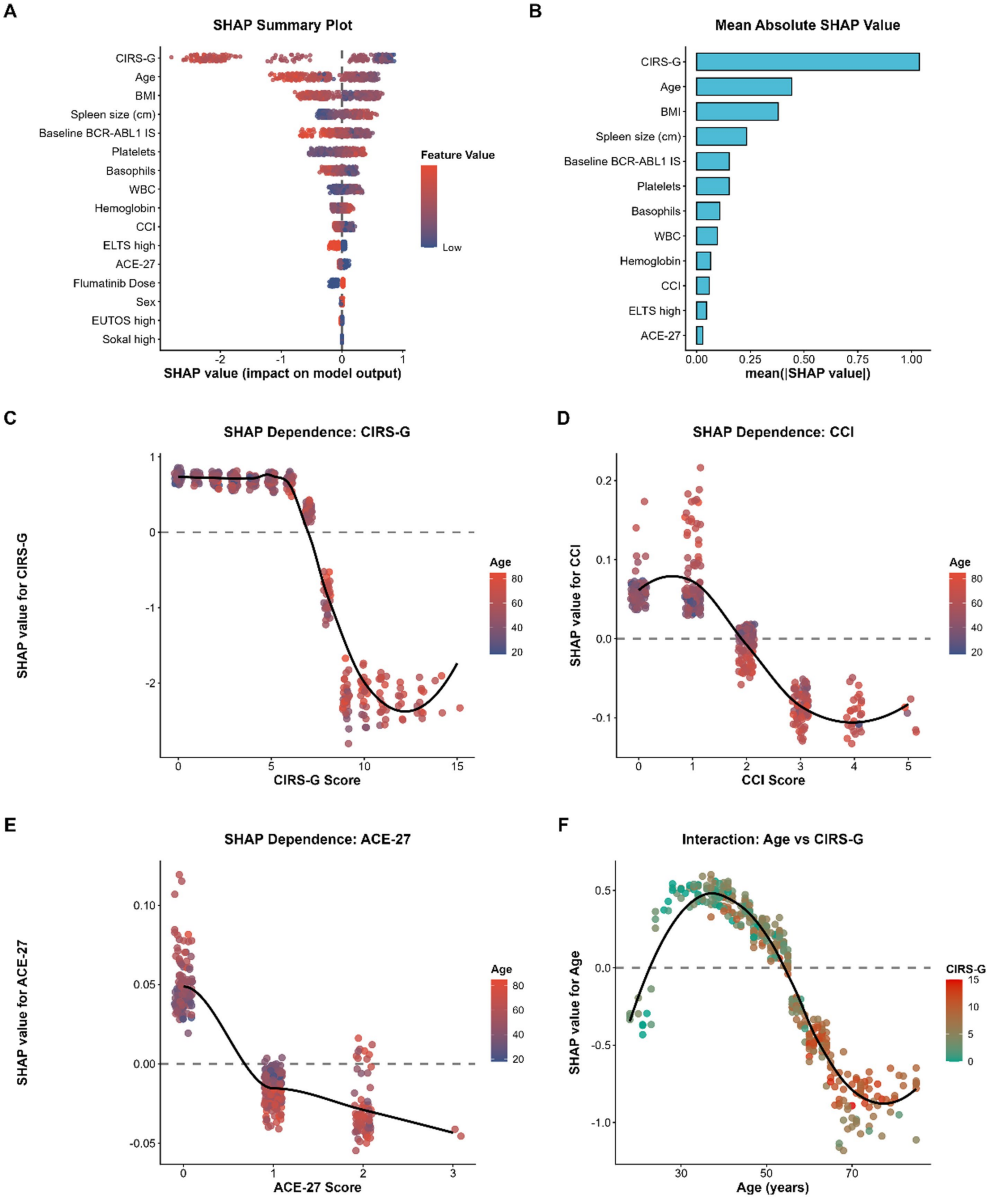
Global SHAP analysis indicating feature impact on MMR prediction. **(A)** SHAP summary plot illustrating the distribution of SHAP values for each feature. **(B)** Mean absolute SHAP values ranking the overall impact of features. **(C–E)** SHAP dependence plots with LOESS smoothing curves showing the potentially non-linear relationships between individual comorbidity scores (CIRS-G, CCI, and ACE-27) and the model’s output. **(F)** Interaction plot suggesting a potential negative synergistic effect between age and CIRS-G scores.

To elucidate the underlying mechanisms by which the XGBoost model generates its predictions, SHapley Additive exPlanations (SHAP) analysis was employed to interpret the global influence and non-linear relationships of the integrated features. The SHAP summary plot allows one to review how distinct features impact a model (Figure 3A). Each feature is represented in the form of a colored dot. The greater the feature value, the more the dot is red. The average CIRS-G score and higher age were found to correspond more often to SHAP values that were negative. This suggests that higher age and more comorbidities are features that correlate to a lower likelihood of attaining MMR. The mean absolute SHAP values are consistent with the Gain-based feature importance rankings. CIRS-G was ranked highest with a mean absolute SHAP value of 1.037 and age, 0.443, BMI, 0.380, and traditional markers of tumor burden like size of the spleen of 0.233 and baseline BCR-ABL1 IS of 0.152 were ranked lowest (Figure 3B).

The particular clinical thresholds pertaining to the comorbidity systems were assessed by creating SHAP dependence plots along with LOESS smoothing curves. A CIRS-G score is a non-linear curve (Figure 3C). The curve indicates that the SHAP values are positive and relatively constant until the CIRS-G score reaches 7, where a notable drop occurs and enters the negative values. This indicates that having a CIRS-G score of 8 or more should be seen in clinical assessment as having significant impact to the likelihood of achieving a molecular response.

In a similar vein, though with marked differences in threshold levels and considerably reduced impact, nonlinear effects were determined for the two other comorbidity scoring systems. For the CCI, the impact decreased and crossed the zero baseline starting at a score of about two (Figure 3D). The impact for ACE-27 switched to negative values at scores of 0.5 and 1 (Figure 3E). This could mean that the three comorbidity scales have different thresholds for explaining outcomes of the treatment.

An interaction plot was also made for a combined effect of age and CIRS-G scores, and the results of the model (Figure 3F). It was seen that the age-related SHAP value exhibited an inverted U-shaped relationship. The expected value was positively high when age was between 35 and 45, and dropped continuously when age was above 50. Particularly, in the age group above 60, high CIRS-G scores (red and brown dots) were observed at the lowest level of the SHAP curve. Thus, there seems to be a negative interaction when age and comorbidity are considered together, since the two factors together seem to determine how poorly effective target therapy was in the high-risk population.

### 3.4 Incremental predictive value and synergistic interaction of comorbidity scoring systems

Having observed the non-linear associations between different comorbidity scores and target therapy outcomes, we further evaluated the incremental predictive value of incorporating each specific comorbidity scoring system into a baseline clinical model. The first baseline XGBoost model was trained using age, baseline tumor burden, and standard laboratory metrics, obtaining an area under the curve (AUC) of 0.725 for 12-month major molecular response (MMR) prediction.

**Figure 4:**
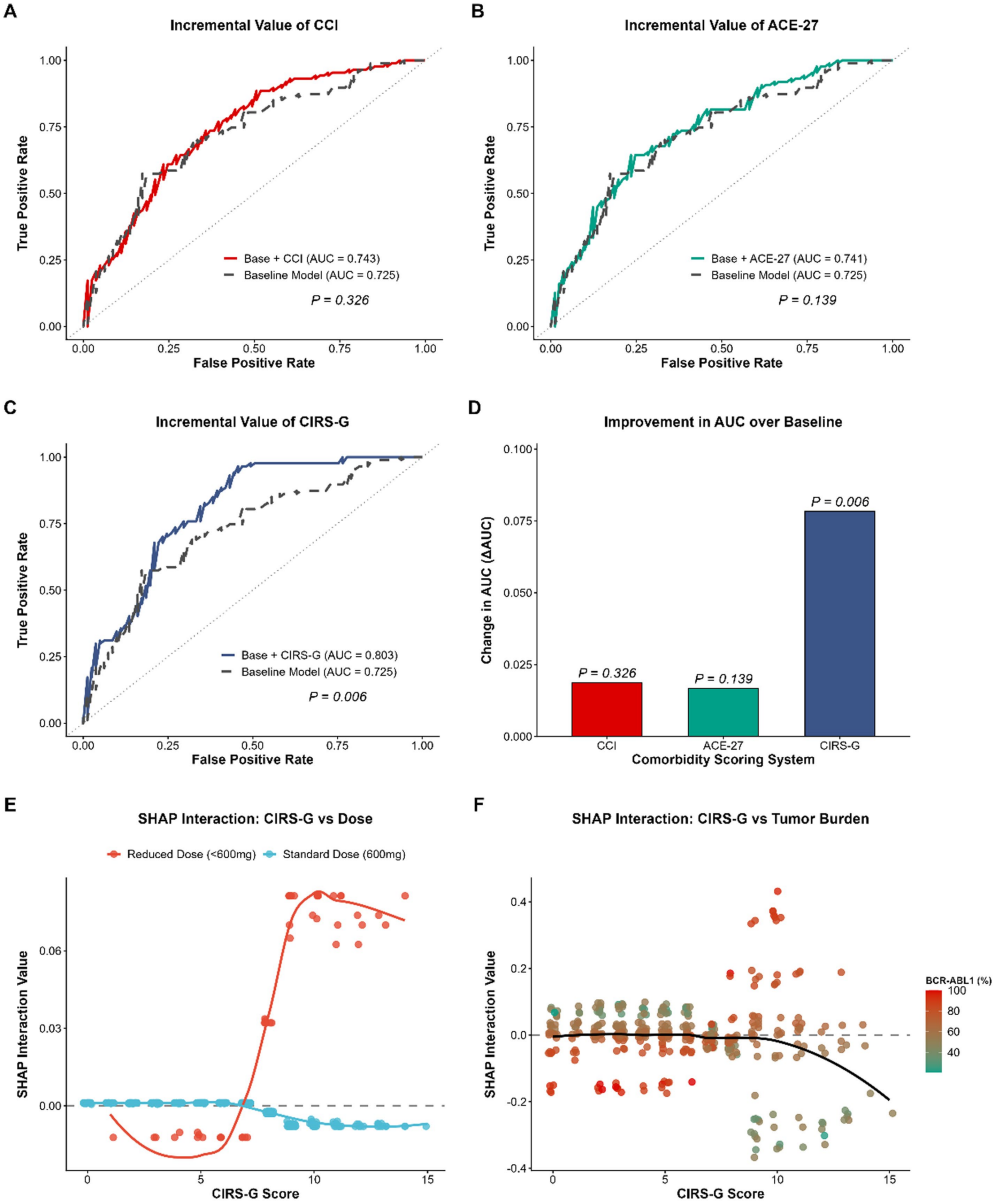
Incremental predictive value of comorbidity scoring systems and SHAP interaction analysis. **(A–C)** ROC curves comparing the baseline clinical model with models incorporating CCI, ACE-27, or CIRS-G, respectively. **(D)** Bar chart showing the change in AUC (ΔAUC) evaluated by DeLong’s test, suggesting the incremental value of CIRS-G over the baseline model. **(E,F)** SHAP interaction plots indicating how CIRS-G scores might interact with initial flumatinib dose and baseline tumor burden (BCR-ABL1 IS) to influence therapeutic outcomes.

The baseline models with CCI (AUC 0.743; Figure 4A) and ACE-27 (AUC 0.741; Figure 4B) only had positive changes of AUC ≤ 0.02, and DeLong’s test showed no statistically significant changes (ΔAUC = 0.019 for CCI, *p* = 0.326; ΔAUC = 0.017 for ACE-27, *p* = 0.139) indicating these metrics had little value with this cohort (Figure 4D). However, inclusion of CIRS-G showed an improved model with AUC = 0.803 (Figure 4C), and this was statistically significant (ΔAUC = 0.078, *p* = 0.006; Figure 4D). These results suggest that CIRS-G captures additional data concerning the patient’s physiological status not captured by clinical metrics.

Owing to its apparent predictive superiority, the CIRS-G variable was selected to pursue SHAP interaction analysis for clarifying its associates along with other clinical measures and baseline disease attributes. SHAP interaction plot for CIRS-G and initial flumatinib dose portrayed differentiated patterns along the various strata of common clinical comorbidities (Figure 4E). For patients with mild to moderate comorbidities (CIRS-G < 7), the interaction SHAP value of the reduced dose was most often negative; conversely, for patients with a comorbidity score of 8 or above, the reduced dose showed a strong positive interaction SHAP value shift. This case illustrated what seems to be a contextual associative phenomenon – the dose reduction could potentially correlate with positive treatment response specifically in the cohort with severe comorbidity.

In addition, interaction analyses for CIRS-G and baseline tumor burden (Baseline BCR-ABL1 IS) were also carried out (Figure 4F). While the CIRS-G score was low, the smoothing curve showed the interaction value to be almost flat. However, steep negative values were noted for interaction SHAP values as CIRS-G score > 10. In addition, a higher baseline tumor burden was represented with red and brown dots, which were noted to co-occur with low values of the interaction. This phenomenon demonstrated a negative interaction, and the severe comorbidity along with a high tumor burden appeared to carry an excess risk of an unsatisfactory molecular response.

### 3.5 Individualized interpretation of the predictive model using local SHAP analysis

**Figure 5:**
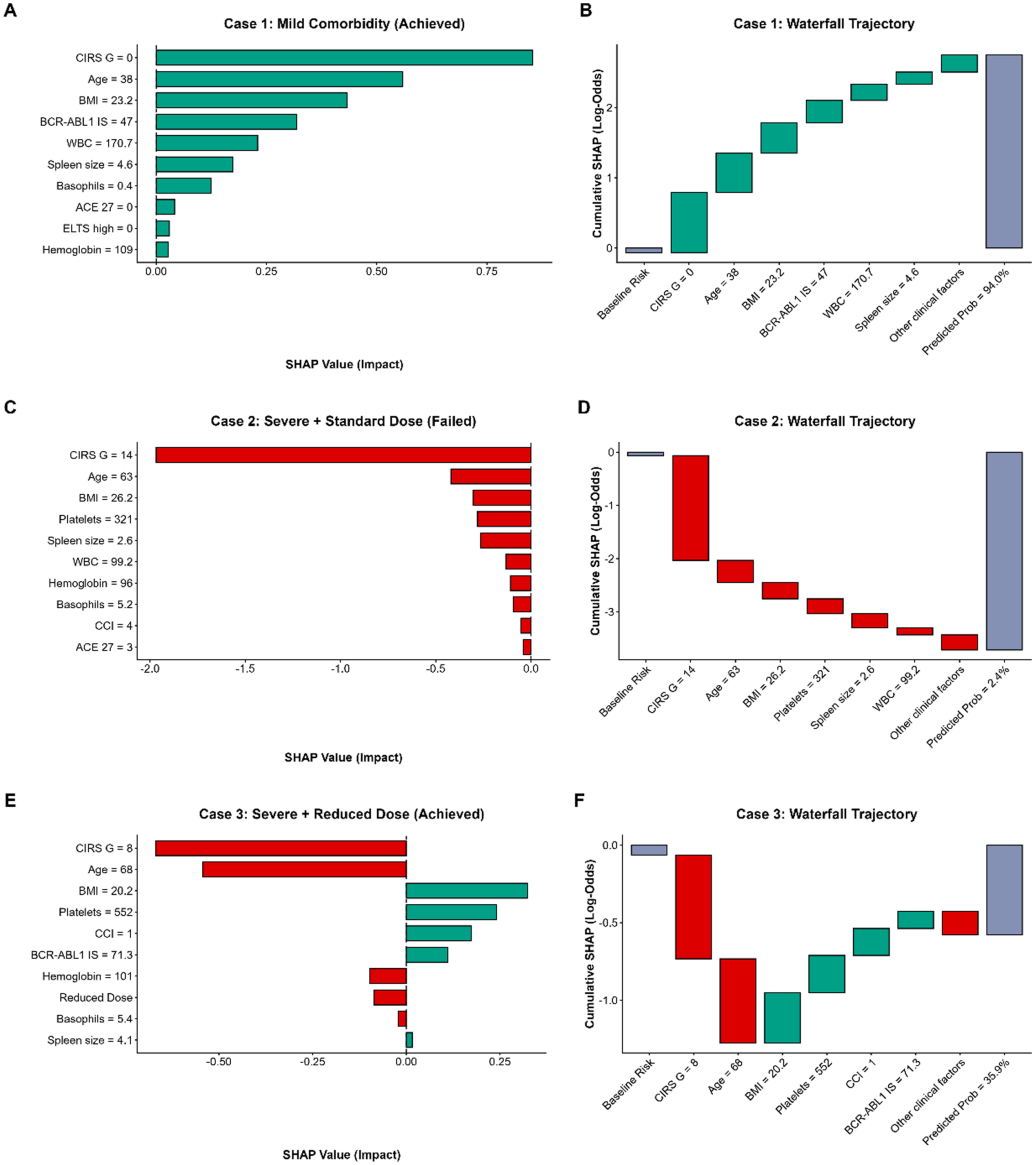
Local SHAP analysis explaining individualized clinical trajectories. **(A,B)** Feature contribution bar chart and waterfall plot for Case 1, representing a patient with mild comorbidity achieving MMR. **(C,D)** Local SHAP explanations for Case 2, a patient with severe comorbidity receiving standard-dose therapy who failed to achieve MMR. **(E,F)** Local SHAP explanations for Case 3, a patient with severe comorbidity receiving a reduced dose who successfully achieved MMR. These individual trajectories suggest that precision dose adjustment might positively influence outcomes in specific high-risk populations.

While global SHAP evaluations revealed overall feature importance and interactions across the cohort, local SHAP analysis was further performed to illustrate how these variables jointly influenced individual clinical predictions. To illustrate the model’s possible applicability in personalized risk evaluation and dose tailoring, we chose three clinical cases from the testing set (Figure 5).

In case 1, we focus on the younger patient (Age = 38 years) who has a mild comorbidity burden (CIRS-G = 0, ACE-27 = 0, CCI = 0). The contributed feature bar chart and waterfall plot both showed a consistent buildup of positive SHAP values (Figures 5A,B). The absence of severe comorbidity (CIRS-G SHAP = +0.854), younger age (SHAP = +0.559) and favorable BMI (23.2, SHAP = +0.433) in conjunction with a baseline BCR-ABL1 IS (47%, SHAP = +0.319) contributed to improved and positive outcomes. All these positive predictors, contributed to a > 90.0% predicted probability of achieving major molecular response (MMR), which matched the patient’s clinical outcome.

Case 2 exemplifies a classic high-risk case due to older age (63 years) and a high comorbidity burden (CIRS-G = 14). The local SHAP analysis showed a particularly high negative contribution (Figures 5C,D). The high CIRS-G score was the most significant negative contributor (SHAP = −1.968) and was accompanied by the older age (SHAP = −0.421) and the higher BMI (26.2, SHAP = −0.303). Given a standard flumatinib dose, the ongoing presence of these negative predictors resulted in a markedly low predicted probability of 2.4%. The presence of high comorbidity and standard dosing in an elderly patient suggested a high likelihood of therapy failure, which is evident in this case.

Case 3 illustrates what might be a case-by-case unique intervention plan. This patient is also considerably older (68 years) and has a severe comorbidity burden (CIRS-G = 8), which generated substantial initial negative SHAP values (CIRS-G SHAP = −0.669, Age SHAP = −0.543; Figures 5E,F). However, unlike Case 2, here, a lower flumatinib dose was prescribed. While this dose, in isolation, has a small negative SHAP value of −0.086, it is possible that, by reducing systemic toxicity, some other positive factors (e.g., an optimal BMI of 20.2 (SHAP = +0.324) and a lower CCI of 1 (SHAP = +0.173) were able to have a positive effect). Considering these clinical factors contributed a lot in the positive prediction of probability (35.9%) and the patient attained MMR. This pathway showed that an individualized dose adjustment in the context of comorbidity profiling could moderate the adverse impact of high CIRS-G scores associated with older patients.

### 3.6 Clinical utility, risk stratification, and robustness of the predictive model

**Figure 6:**
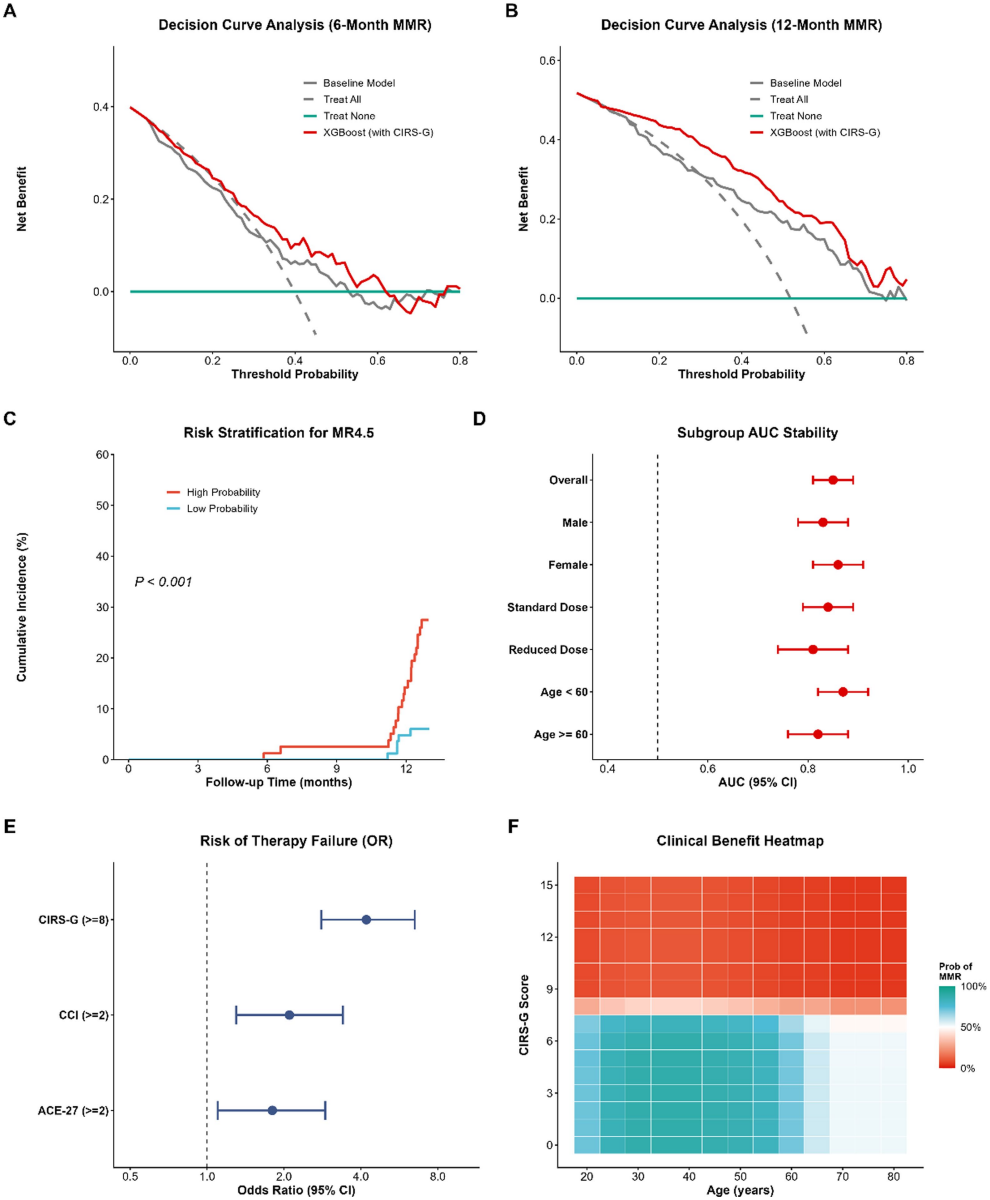
Assessment of clinical utility and risk stratification of the proposed model. **(A,B)** Decision curve analysis (DCA) for 6-month and 12-month MMR, indicating that the CIRS-G-integrated XGBoost model may offer a potential net clinical benefit. **(C)** Kaplan–Meier curves demonstrating risk stratification for achieving MR4.5 based on the model’s predicted probabilities. **(D)** Forest plot showing the stability of the model’s AUC across different clinical subgroups. **(E)** Forest plot comparing the odds ratios (OR) for therapy failure among patients with severe scores across the three comorbidity systems. **(F)** Clinical benefit heatmap suggesting the joint probabilistic impact of age and CIRS-G score on MMR.

To evaluate the potential clinical utility of the proposed predictive framework, Decision Curve Analysis (DCA) was conducted. The DCA curves for 6-month and 12-month MMR projections showed the XGBoost model with the CIRS-G score yielding a greater net clinical benefit over most mean threshold probabilities than the other clinical model and the “treat-all” and “treat-none” models (Figures 6A,B). This finding indicated that utilizing the CIRS-G-integrated machine learning model for therapeutic decision-making—where a high-risk prediction would explicitly trigger clinical actions such as closer molecular monitoring, proactive management of concomitant illnesses, or early consideration of dose optimization—might yield improved net clinical benefits for patients.

Using model-generated probabilities, patients in the testing cohort were divided into high and low groups based on the median probability threshold. Kaplan–Meier analysis showed a 12-month significant difference (*p* < 0.001; Figure 6C) in deep molecular response (MR4.5) achievement. The high-probability group exhibited a substantially higher rate of reaching MR4.5, suggesting that the model could be a valuable tool for identifying patients with favorable long-term target therapy responses.

Stability regarding the model’s predictive performance was evaluated in several clinical subgroups (Figure 6D). The forest plot showed the AUC values were relatively consistent in the different demographic and treatment groups such as sex (Male: 0.83; Female: 0.86), and flumatinib dose (Standard: 0.84; Reduced: 0.81), as well as in the age groups (Age < 60: 0.87; Age ≥ 60: 0.82). The overall AUC was 0.85 (0.81–0.89), which means that the predictive capability of the model was consistent and was not significantly impacted by the variations within subgroups.

Furthermore, a univariable odds ratio (OR) analysis was conducted to examine the risk of therapy failure among patients with high comorbidity scores across the three scoring systems (Figure 6E). To address potential collinearity among clinical covariates and confirm whether CIRS-G provides independent prognostic value rather than acting merely as a proxy for age, we further performed a multivariable logistic regression analysis using Firth’s penalized likelihood method (Supplementary Figure S2). This model adjusted for chronological age, sex, BMI, baseline disease burden, CML risk scores, initial flumatinib dose, and all three comorbidity indices simultaneously. The multivariable model demonstrated that a severe CIRS-G score (≥8) remained an independent and highly significant predictor of 12-month therapy failure, whereas chronological age did not maintain independent statistical significance. The analysis showed that among patients with a CIRS-G score ≥ 8, the risk of therapy failure was the highest (OR = 4.2, 95% CI: 2.8–6.5), and this risk was significantly greater compared to patients with a CCI score ≥ 2 (OR = 2.1, 95% CI: 1.3–3.4) and an ACE-27 score ≥ 2 (OR = 1.8, 95% CI: 1.1–2.9).

To assist with clinical usage, a clinical benefit heatmap was generated using the model output (Figure 6F). The heatmap combined age and CIRS-G scores to show the predicted success rate of achieving MMR. The pattern distributed showed the likely success of the treatment was higher in younger patients with lower CIRS-G scores (highlighted in green and blue regions). Regardless of the age group, if the CIRS-G score was 8 or higher, the predicted success rate was significantly lower (indicated by the uniform red blocks). This visualization proposed a potential “threshold effect” in the case of severe comorbidities - a high comorbidity burden appears to be the primary limiting factor affecting the target therapy, outweighing the influence of age. Furthermore, to address potential temporal bias spanning the six-year inclusion period, a time-based sensitivity analysis was conducted (Supplementary Figure S1). Evaluated strictly within the independent testing set, the XGBoost model demonstrated robust discriminative power across different calendar periods, achieving an AUC of 0.803 for patients treated in the early phase (2018–2020) and 0.894 for those in the late phase (2021–2024) (Supplementary Figure S1A). Calibration curves also remained stable across both phases (Supplementary Figure S1B), confirming acceptable temporal validity.

## 4 Discussion

The advent of targeted therapies such as flumatinib has significantly improved the prognosis of patients with chronic-phase chronic myeloid leukemia (CP-CML), yet the increasing prevalence of comorbidities in an aging patient population complicates therapeutic management (22). Machine learning and SHAP analysis have been applied for the first time to assess the predictive utility of three comorbidity scoring systems (CCI, ACE-27, and CIRS-G). CIRS-G demonstrates the most incremental predictive value regarding molecular response and shows intricate relationships with age and starting drug dosage.

The burden of comorbidity has been shown to adversely influence the clinical effectiveness of tyrosine kinase inhibitors (TKI) and the adherence of CML patients to their treatment (23). In agreement with these findings, our study indicates that CIRS-G is the most predictive of 12-month major molecular response (MMR) compared to the CCI and ACE-27 systems (24–26). This is likely related to the structural design of these tools. CCI is primarily concerned with predicting mortality, and ACE-27 is focused on certain solid tumor situations, while CIRS-G is a broad assessment of multiple organ systems, which likely captures a physiologic decline due to the aging process more so than the other two systems. A design element of this study is whether CIRS-G simply acts as a proxy for age, and biologically there is a correlation. However, CIRS-G record a decline in physiologic reserve and age rather than solely time as demonstrated in the AUC and interaction analyses.

After the application of the XGBoost algorithm alongside SHAP (SHapley Additive exPlanations) analysis, we were able to pinpoint the presence of certain non-linear associations in our dataset, which, to our knowledge, had not yet been quantified. These associations are often missed by traditional statistical methods due to their inherent limitations. XGBoost coupled with SHAP helps in detecting shifts/stages (clinical cut-off/threshold) that are likely to define a certain disease/clinical composite condition, especially when the condition it describes is organ dysfunction (27, 28). From the SHAP plots, we define a threshold to the CIRS-G where with the score of 8 or above (CIRS-G) a patient is predicted to reach a significant decline in the MMR probability. This observation from the SHAP plots likely represents a critical clinical threshold, where the cumulative exhaustion of organ systems becomes the dominant prognostic factor.

The exploratory interaction analysis between initial flumatinib dose and CIRS-G scores reveals a model-based association, wherein dose reduction correlates with altered predictive outputs for patients with CIRS-G ≥ 8. Because dose reduction was not randomly assigned and likely reflects clinician judgment regarding patient frailty and toxicity, this SHAP-derived pattern cannot establish a causal relationship. Rather, it highlights a complex associative structure that aligns with real-world observations regarding dose management in frail CML patients, warranting rigorous prospective investigation (29, 30). Additionally, the clinical benefit heatmap illustrates that severe comorbidity (CIRS-G ≥ 8) might exert a dominant prognostic effect, which is greater than the survival benefit typically conferred by a younger age in isolation. This is vital in expressing the need for a comprehensive assessment of the patient before the initiation of the therapeutic intervention (31).

This study has a number of limitations that must be addressed. First, the retrospective, singular-center design inherently carries selection bias and limits the generalizability of the predictive model. Furthermore, because the dataset spans six calendar years and relies on a random train-test split, temporal variations in supportive care protocols and monitoring intensity were mixed. Consequently, the reported area under the curve may overestimate true prospective performance, underscoring the necessity for future time-based external validations across multiple centers. Second, the existing dataset lacks finer, time-based, and granular data on specific adverse events and dose interruptions, which may be a connective mechanism, especially when certain severe comorbidities are associated with low molecular responses. Third, flumatinib possesses a specific efficacy and toxicity profile and is primarily utilized in China. The findings of this study cannot be directly extrapolated to patients treated with other TKIs (such as imatinib, dasatinib, or nilotinib), which interact differently with specific comorbidities. External validations in independent cohorts across diverse TKI regimens are imperative before any definitive clinical recommendations can be formulated. Furthermore, calculating the CIRS-G score requires structured assessment across multiple organ systems, making it inherently sensitive to the variable quality of clinical documentation in a retrospective chart review, which may introduce documentation bias.

## 5 Conclusion

This study demonstrates that the integration of the Cumulative Illness Rating Scale for Geriatrics (CIRS-G) into machine learning algorithms provides valuable prognostic information for predicting molecular responses in patients with chronic-phase chronic myeloid leukemia treated with flumatinib. Our analysis suggests that CIRS-G outperforms the Charlson Comorbidity Index and the Adult Comorbidity Evaluation-27 in evaluating comorbidity burden, exhibiting non-linear threshold effects on therapeutic efficacy. Furthermore, SHAP interpretation reveals that severe comorbidities and advanced age may exert negative synergistic effects, which might be partially mitigated by individualized dose optimization. While these findings offer practical insights for precision medicine, the single-center retrospective design necessitates further validation. Future multi-center prospective studies are warranted to explore the dynamic impact of comorbidity-guided dose adjustments on long-term clinical outcomes.

## Data Availability

The raw data supporting the conclusions of this article will be made available by the authors, without undue reservation.
