# Supplementary material for: Evaluating comorbidity scoring systems for flumatinib therapy in chronic myeloid leukemia: a machine learning and SHAP-based predictive analysis
Source: Front Med (Lausanne). 2026 May 28;13:1849735. doi: 10.3389/fmed.2026.1849735 (PMC13253388; doi:10.3389/fmed.2026.1849735)
Supplement: Supplementary file 2 [file Table_1.DOCX]

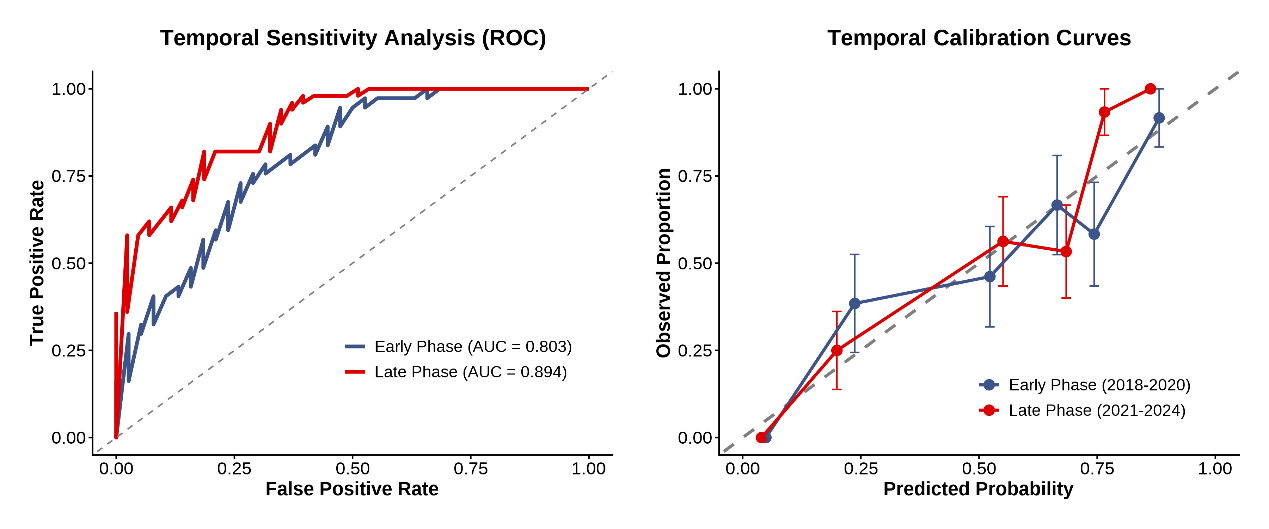


**Supplementary Figure S1. Temporal sensitivity analysis of the XGBoost predictive model across different calendar periods.**

(A) Receiver Operating Characteristic (ROC) curves demonstrating the discriminative performance of the XGBoost model evaluated separately on the independent testing set, stratified into an Early Phase cohort (patients treated 2018–2020) and a Late Phase cohort (patients treated 2021–2024). (B) Temporal calibration curves comparing the model-predicted probabilities of achieving 12-month major molecular response (MMR) with the observed clinical proportions in both the Early Phase and Late Phase cohorts. Error bars represent standard errors.


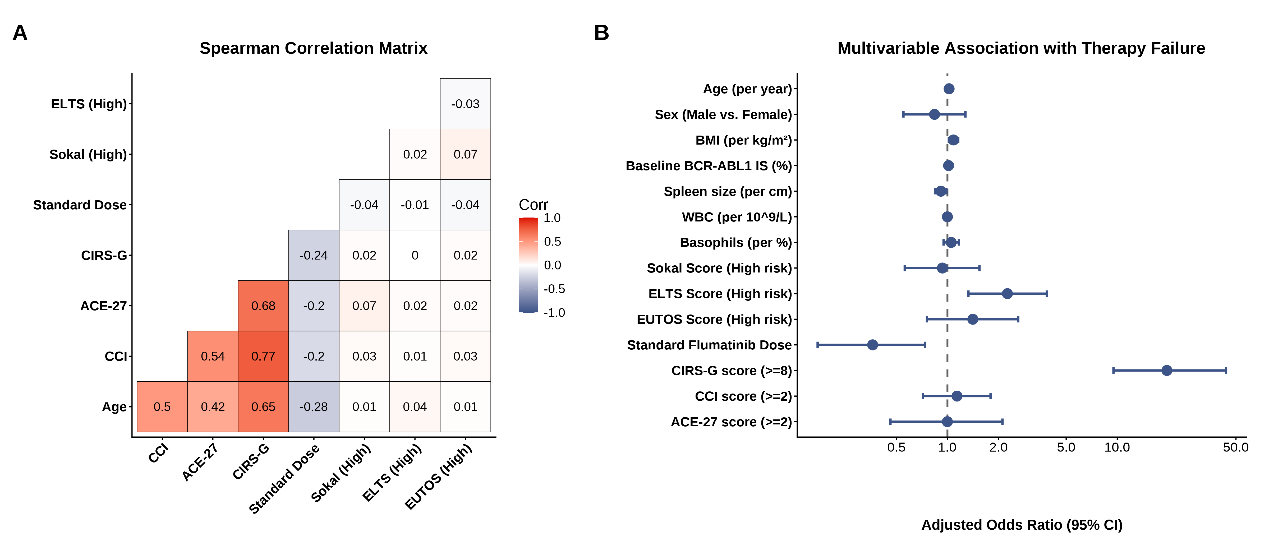


**Supplementary Figure S2. Assessment of collinearity and independent prognostic value of clinical covariates.**

(A) Spearman correlation matrix evaluating potential collinearity among patient chronological age, the three comorbidity scoring systems (CCI, ACE-27, CIRS-G), initial flumatinib dose (standard 600mg vs. reduced dose), and established CML prognostic risk scores (Sokal, ELTS, EUTOS). Correlation coefficients are displayed within the tiles. (B) Forest plot presenting the adjusted Odds Ratios (aOR) and 95% Confidence Intervals (CI) derived from a multivariable logistic regression model using Firth's penalized likelihood. The model predicts 12-month therapy failure (defined as not achieving major molecular response) by simultaneously adjusting for age, sex, BMI, baseline disease burden markers (BCR-ABL1 international scale [IS], spleen size, WBC, and basophils), CML prognostic risk categories, initial dose, and severe comorbidity scores across the three scoring systems (CIRS-G ≥ 8, CCI ≥ 2, and ACE-27 ≥ 2).
